# Association Between eHealth Literacy in Online Health Communities and Patient Adherence: Cross-sectional Questionnaire Study

**DOI:** 10.2196/14908

**Published:** 2021-09-13

**Authors:** Xinyi Lu, Runtong Zhang

**Affiliations:** 1 School of Management and E-business Zhejiang Gongshang University Hangzhou China; 2 School of Economics and Management Beijing Jiaotong University Beijing China

**Keywords:** online health communities, OHCs, eHealth literacy, patient adherence, health information, physician-patient communication

## Abstract

**Background:**

eHealth literacy is significantly associated with patients’ online information behavior, physician-patient relationship, patient adherence, and health outcomes. As an important product of the internet, online health communities (OHCs) can help redistribute idle medical resources, increase medical resource utilization, and improve patient adherence. However, studies on eHealth literacy in OHCs are limited. Therefore, this study examined patients’ eHealth literacy regarding health information–seeking behavior and physician-patient communication in OHCs.

**Objective:**

This study aimed to investigate the association between eHealth literacy in OHCs and patient adherence by employing social cognitive theory.

**Methods:**

This was an empirical study, in which a research model consisting of 1 independent variable (patients’ eHealth literacy), 3 mediators (physician-patient communication in OHCs, patient health information–seeking behavior in OHCs, and patients’ perceived quality of health information in OHCs), 1 dependent variable (patient adherence), and 4 control variables (age, gender, living area, and education level) was established to examine the associations. Multi-item scales were used to measure variables. An anonymous online survey involving 560 participants was conducted through Chinese OHCs in July 2018 to collect data. Partial least squares and structural equation modeling were adopted to analyze data and test hypotheses.

**Results:**

The survey response rate was 79.6% (446/560). The reliability, convergent validity, and discriminant validity were acceptable. Age, gender, living area, and education level were positively associated with patient adherence, and gender was positively associated with physician-patient communication and patients’ perceived quality of internet health information in OHCs. Patients’ eHealth literacy was positively associated with patient adherence through the mediations of physician-patient communication, internet health information–seeking behavior, and perceived quality of internet health information in OHCs.

**Conclusions:**

Results indicate that physician-patient communication, internet health information–seeking behavior, and the perceived quality of internet health information are significantly associated with improving patient adherence via a guiding of eHealth literacy in OHCs. These findings suggest that physicians can understand and guide their patients’ eHealth literacy to improve treatment efficiency; OHCs’ operators should this strengthen the management of information quality, develop user-friendly features, and minimize the gap between the actual and perceived information quality.

## Introduction

### Background

Health literacy is defined as “the degree to which an individual has the capacity to obtain, communicate, process, and understand basic health information and services to make appropriate health decisions,” [1] but individual’s skill to obtain and use health information needs to be redefined with the development of medical internet services [2]. Accordingly, eHealth literacy has emerged [3], which is defined by Norman and Skinner [4] as individuals’ ability to “seek, find, understand, and appraise health information from electronic sources and apply the knowledge gained to addressing or solving a health problem.” eHealth literacy is related to health literacy, and both of them involve the skills of seeking, appraising, and applying health information [5]. Gilstad [6] added the communication of health information into the definition of eHealth literacy, while Neter and Brainin [5] and Paige et al [7] suggested that eHealth literacy is associated with health-related behaviors, communication skills with physicians, self-management, and health outcomes.

In recent years, online health communities (OHCs) have provided the public with new platforms to obtain health information, share medical experiences, and communicate with physicians. OHCs can help redistribute idle medical resources, improve medical resource utilization, and enhance the physician-patient relationship [8-10]. Patients’ eHealth literacy in OHCs has become an important topic to be studied. Previous studies show that patients with adequate eHealth literacy have a higher level of ability to seek internet health information and are more likely to obtain reliable and high-quality health information online than are patients with inadequate eHealth literacy [11,12]. In addition, a high level of eHealth literacy can enable patients to communicate with physicians and improve communication efficiency [11,13].

Physician-patient communication and health information–seeking behavior in OHCs are related to patient adherence, which is defined as “the extent to which a person’s behavior (in terms of taking medications, following diets, or executing lifestyle changes) coincides with medical or health advice” [14-16]. Given that physicians cannot constantly participate in patients’ daily life, the ability of self-management and self-monitoring is important for patients to maintain a healthy lifestyle. Treatments, medical regimens, and therapies are more likely to be effective if patients take medication in accordance with prescriptions and physicians’ advice [17]. Therefore, we speculate that patients’ eHealth literacy is likely to be associated with their adherence. Previous studies have focused on the relationship between health literacy and patient adherence or between OHCs and patient adherence [18,19]. However, studies on the association between eHealth literacy in OHCs and patient adherence are limited because of the short development time of medical internet services, OHCs, and eHealth literacy. Given the low level of medical resource utilization, uneven medical resource distribution, and serious hospital congestion in China, this study aims to identify the association between patients’ eHealth literacy in OHCs and patient adherence through empirical study and by considering physician-patient communication, internet health information–seeking behavior, and the perceived quality of internet health information as mediators. We hope we can propose productive ideas for improving patient adherence that can ultimately alleviate these aforementioned problems. This study can enrich the theoretical study of OHCs, eHealth literacy, and patient adherence, and may have practical implications for managing OHCs and improving patient adherence through eHealth literacy in OHCs.

### Patient Adherence

Patient adherence plays a vital role in health management and health care, especially for patients with chronic diseases [20]. Horwitz et al [20] proved that patient adherence is positively associated with health outcomes and that patients who adhere strictly to medical regimens and physicians’ suggestions are relatively healthier than those who do not. Previous studies have confirmed that low adherence may lead to serious consequences for patients, the economy, and society. For patients with low adherence, diseases may be less likely to be controlled or cured, thereby increasing the morbidity and mortality [21]. For the economy, if patients do not adhere to medical regimens or physicians’ advice, therapies cannot achieve the intended outcomes, which may waste medical resources [22,23]. In terms of social effect, the unbalanced medical resource allocation may be serious. For example, medical resources may be wasted in some hospitals in economically developed regions, while hospitals in some regions may lack sufficient resources. Moreover, some genuinely beneficial drugs may be mistaken as useless and then be discontinued because patients do not take medications in accordance with prescriptions [23].

Given that patient adherence is a dynamic parameter [24], patient adherence can be enhanced to improve patients’ outcomes, increase the utilization of medical resources, advance the clinical testing of drugs, and promote the stable development of society.

### Online Health Communities

This study mainly discusses the OHCs in which patients and physicians participate. Patients are increasingly using OHCs to seek health information and communicate with physicians about medical and emotional support because of certain benefits brought by OHCs [25,26]. First, patients can ask questions and communicate with physicians by creating posts and sending online messages anytime and anywhere without visiting hospitals [27]. Moreover, users can conveniently access OHCs’ corresponding apps using smartphones. Second, patients’ privacy can be protected as meeting face-to-face is unnecessary, and OHCs generally allow patients to ask questions anonymously [28]. Third, OHCs can help alleviate hospital congestion and integrate idle medical resources [9,10,15]. Specifically, patients can diagnose a few basic and simple symptoms by themselves based on the support from OHCs and do not need to visit hospitals frequently or wait extensively. In turn, physicians can spend their time communicating with patients in OHCs if there are only a few patients present in hospitals. In this way, medical resource utilization can be improved.

However, several drawbacks of OHCs must be considered. The observation of patients’ breath, sound, and facial expressions is important for physicians in diagnosing illness accurately [29], which is difficult to achieve though OHCs. In this type of situation, physicians may misunderstand patients’ conditions and provide inaccurate advice, which may decrease patients’ satisfaction. In addition, some patients may worry that they cannot receive answers from actual physicians. Given OHCs’ advantages and persistent development, studies on OHCs should be conducted to decrease or avoid these shortcomings.

### eHealth Literacy

eHealth literacy is nested in the context of a digital environment. Based on Norman and Skinner [4], Gilstad [6] extended the definition of eHealth literacy to include an individual’s skill in identifying, defining, and solving a health problem by communicating, seeking, understanding, appraising, and applying health information and digital technologies under a given cultural, social, and situational background [30]. Paige et al [31] further proposed a transactional model to update the definition of eHealth literacy to include a patient’s skill in interacting with the external environment and in counteracting the negative effects of environmental factors. eHealth literacy is a central skill [31] and is significantly associated with health-related behavior [11,32], self-management skills, patients’ health-related decision-making, and health outcomes [33,34]. For example, adequate eHealth literacy can help patients understand their conditions accurately, obtain health-related information and knowledge, and improve the ability to self-manage their health and communicate with physicians [11]. In addition, eHealth literacy is associated with health responsibility, self-actualization, and the relationship between patients and health care providers [35].

Previous studies have verified that patients with a high level of eHealth literacy are good at seeking, selecting, and assessing health information from many sources using additional search strategies [5,32], whereas patients with limited eHealth literacy may find the use of online health-related resources difficult. Freemann et al [36] asserted that eHealth literacy plays an important role in adolescents’ daily life. Mitsutake et al [32] found that eHealth literacy can promote individuals’ health behaviors, such as physical exercise and balanced nutrition. Bodie and Dutta [37] suggested that eHealth literacy can help develop patients’ ability to solve specific health problems by themselves using information obtained from the internet, thereby possibly encouraging patients’ health behavior. In this study, we focused on patients’ eHealth literacy in OHCs. Specifically, we concentrated on patient behavior in seeking, obtaining, understanding, and evaluating health-related information in OHCs and the association between eHealth literacy and patient adherence.

### Internet Health Information

In terms of content, health information can be divided into 2 categories: health care information and healthy lifestyle information [5,38,39]. The internet has become the main source for individuals to seek health information [40], and various institutions, such as governments, medical institutions, and business corporations, have established health-related websites to provide information-seeking platforms for the public [41]. According to Wilson [42], combined with the context of the internet, internet health information behavior can be categorized as internet health information–seeking behavior, internet health information use behavior, online communication behavior, and passive receiving of internet health information behavior. According to Neter et al [43], individual’s online behaviors in the context of Web 1.0 are mainly consumption activities, such as reading others’ experiences, watching videos, and sending or receiving emails, while behaviors in the context of Web 2.0 are mainly production activities, such as rating physicians and hospitals, communicating with others, and sharing their experiences. The aim of individual’s use of OHCs is obtaining health information, which indicates that this behavior is active. This study intends to identify the association between eHealth literacy in OHCs and patient adherence. eHealth literacy requires the skill of seeking health information from digital sources. However, having a capacity to seek information online does not mean that the individual would like to perform health information–seeking behavior. Rather, eHealth literacy may be associated with an individual’s internet health information–seeking behavior. Therefore, we mainly focused on the internet health information–seeking behavior and online communication behavior in OHCs, which includes Web 1.0 and Web 2.0 health-related activities.

Given the zero gatekeeping and zero-cost publishing of the internet, information can be published and spread quickly and in timely fashion [44], and patients can obtain health information conveniently through the internet, which may help improve their ability in health-related problem-solving [45]. However, in consideration of the universal accessibility of the internet, internet health information has several shortcomings, such as the quality of information [46]. For one, each person is allowed to publish health information online regardless of its correctness, as, for example, most websites in China lack a strict review mechanism for health information. For another, individuals may have inadequate eHealth literacy to appraise the quality of health information or select reliable facts [5]. With limited eHealth literacy, individuals are unable to accurately appraise health information and may at times regard actual high-quality information as low quality or regard actual low-quality information as high quality. For example, a person who does not have adequate eHealth literacy may not be able to find reliable health information from online sources, but sometimes he or she may consider the information obtained from an official health portal as high quality. Therefore, patients’ eHealth literacy may be associated with their perceived quality of internet health information, and thus this study concentrated on patients’ perception of internet health information quality in OHCs.

### Physician-Patient Communication

Originally, the communication between physicians and patients could only occur in hospitals. A new form of physician-patient communication has emerged with the internet, and OHCs serve as important platforms. Because of the advantages of OHCs, patients are willing to communicate with physicians online before or after visiting hospitals [25,47], which is beneficial for improving the efficiency of offline treatments and saving time. Patients can initially assess their conditions through communication with physicians in OHCs before visiting hospitals, which may help them select the relevant health care department and minimize unnecessary checks [48]. In addition, communicating with physicians in advance can help patients gain information of physicians and decrease the uncertainty in offline treatments [10].

The internet can provide more opportunities to communicate [49]. It is better to practice individuals’ communication skills through online channels than on offline channels. Therefore, online communication may be more effective than offline communication. To specify, without a face-to-face meeting, patients and physicians do not need to answer immediately. Therefore, they have adequate time to reconsider their responses, and patients’ sense of unfamiliarity and nervousness can be reduced. In addition, conflicts between physicians and patients may be avoided in OHCs, thereby improving communication efficiency and quality and promoting patients’ satisfaction with physicians [45].

### Model and Hypotheses

**Figure 1:**
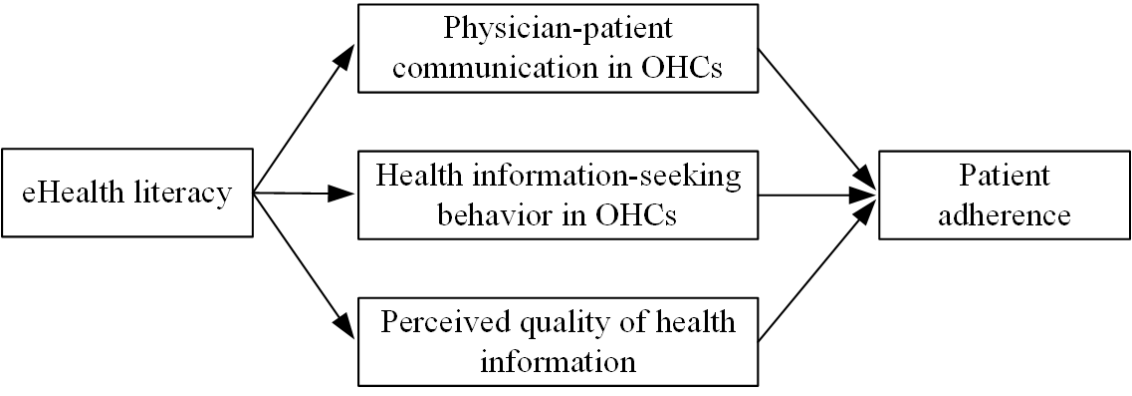
Research model. OHC: online health community.

Human behavior can be associated with social information obtained from their environment [50]. Specifically, individuals can obtain information from their social environment when they lack knowledge, which may influence their opinions, beliefs, and behavior [51]. In the context of this study, patients actively obtain health information from OHCs by communicating with physicians and searching for information to satisfy their demand for health information and to improve their self-efficacy, which may be associated with patient health-related behavior. According to the review by Neter and Brainin [5], eHealth literacy is associated with individuals’ health behaviors. Therefore, we established a research model (Figure 1), which involves 1 independent variable, 3 mediators, and 1 dependent variable to identify the association between eHealth literacy in OHCs and patient adherence through the mediations of physician-patient communication, patient health information–seeking behavior, and patients’ perceived quality of health information by employing social cognitive theory. Social cognitive theory can be used to predict health-related behaviors [52,53]. Self-efficacy is a critical factor in social cognitive theory [54], and it can influence an individual’s way of thinking, thereby self-regulating motivations of behavior. Specifically, a high level of self-efficacy can encourage individuals to change their behavior, while a low level of self-efficacy may make individuals feel frustrated and refuse to change.

Communication is an important skill related to eHealth literacy [5,6], and patients’ communication behaviors may be associated with individuals’ eHealth literacy. Patients with limited eHealth literacy may not have favorable information communication skills and may not be able discuss their illnesses and conditions with physicians clearly [55]. The lack of eHealth literacy may decrease patients’ self-efficacy in communication with physicians in OHCs. According to social cognitive theory [54], patients’ intention to communicate with physicians may be reduced and they may not want to communicate with physicians through OHCs. We thus assumed that eHealth literacy is associated with communication between physicians and patients in OHCs and proposed the following hypothesis: H1*:* Patients’ eHealth literacy is positively associated with physician-patient communication in OHCs.

Seeking health information is another way for patients to obtain health information in OHCs, and it may be associated with eHealth literacy. First, patients with limited eHealth literacy may not be familiar with OHCs. Even if these patients realize the existence of OHCs, they may not be aware that health information can be searched for through these portals [56]. Second, patients with low eHealth literacy may not be good at evaluating the quality of information obtained from OHCs [12], which may decrease patients’ intention to seek health information online. Furthermore, patients with inadequate eHealth literacy may find it difficult to obtain high-quality health information. Third, inadequate eHealth literacy makes patients lack adequate confidence in seeking suitable health information in OHCs [11]. Accordingly, under the guidance of social cognitive theory [54], patients with limited eHealth literacy may have low a level of self-efficacy in seeking health information through OHCs, which may inhibit their information-seeking behavior in OHCs. We thus proposed the following hypotheses: H2*:* Patients’ eHealth literacy is positively associated with patients’ internet health information–seeking behavior in OHCs; H3*:* Patients’ eHealth literacy is positively associated with patients’ perceived quality of internet health information in OHCs.

Patients’ attitudes and behaviors can be associated with health information obtained from OHCs [50]. Therefore, we assume that, as two ways for patients to obtain health information in OHCs, communicating with physicians and seeking health information are associated with patient adherence. The physician-patient relationship can be enhanced through effective communication, which may promote information processing and decision-making [57,58]. Therefore, patients are likely to perceive high-quality health information and consider their physicians to be professional [59], ultimately improving their self-efficacy. A high level of self-efficacy can encourage patients to adopt health-related behavior [54], such as adherence with physicians’ advice. Indeed, Roberts et al [60] confirmed that communication between patients and physicians can improve patient adherence. In terms of health information–seeking in OHCs, generally, the information obtained by patients in OHCs is often basic health-related knowledge, such as medical terminologies and diagnoses, which has been already grasped by physicians [47]. When patients are aware of that fact, they are more likely to trust in their physicians’ professional competence. Thus, patients may be increasingly willing to follow the regimens and advice given by physicians. These considerations produce the following hypotheses: H4*:* Physician-patient communication in OHCs is positively associated with patient adherence; H5: Patients’ internet health information–seeking behavior in OHCs is positively associated with patient adherence; H6: Patients’ perception of internet health information quality is positively associated with patient adherence.

In addition, control variables (age, gender, living area, and education level) were included in this research model to examine and adjust the effects of demographic factors on the research model.

## Methods

### Instrument Development

The survey instrument (see Multimedia Appendix 1) was developed on the basis of scales validated by previous studies. Specifically, eHealth literacy was measured using a 10-item scale consisting of 8 main items adopted from Norman and Skinner [61] and 2 supplementary items adopted from Park and Lee [62]. Physician-patient communication was measured using a 14-item scale adopted from Makoul et al [63]. A 4-item health information–seeking scale reflects the means by which people search and apply health information actively and passively [64]. Laugesen et al [47] adopted a 16-item scale to measure the perceived quality of internet health information and a 5-item scale to measure patient adherence.

We conducted a formal investigation of Chinese individuals who had communicated with physicians and sought health information in Chinese OHCs. Therefore, the questionnaire was translated into Chinese. The translation process was divided into 3 stages [65,66]. First, 3 native Chinese speakers who had at least a master’s degree in English and were skilled in scientific research translation were recruited to translate the instrument into Chinese. Second, we conducted a pretest to acquire advice for improving the comprehensibility, conciseness, readability, and cross-cultural adaptation of the instrument. Third, we recruited English professionals to translate the Chinese questionnaire back to English and to compare the final English version with the original English version to guarantee the conceptual consistency between the 2 versions.

### Data Analysis

Our participants were those who had obtained health information by communicating with physicians and seeking health information in OHCs within the previous month. Thus, they could recall relevant experiences. The formal survey was anonymously conducted through several Chinese OHCs in July 2018, and we guaranteed that the privacy of participants would be strictly protected.

As a comprehensive and nonparametric structural equation modelling (SEM) approach, partial least squares (PLS)-SEM is effective in analyzing complex models, providing robust model estimations, and evaluating the quality of predicting results [67-69], especially in the case of complex models or small sample sizes. In addition, PLS-SEM can be used to conduct mediation analysis [68]. Therefore, this study used PLS-SEM to test hypotheses and analyze the research model [47,68] and adopted SmartPLS software version 3.2.8 to analyze data. First, we re-evaluated the reliability and validity of scales since the research context and participants in this study were different from previous works. Specifically, we calculated Cronbach α to assess the reliability and adopted confirmatory factor analysis to assess the convergent validity and discriminant validity. Second, we calculated the magnitude and significance of path coefficients to examine the effects of control variables and test hypotheses, and used multivariate coefficient of determination (*R*^2^) to calculate Cohen *ƒ*^2^ and used goodness of fit (GoF) to analyze the effect sizes of the research model and the fit between the research model and observed data [70]. Third, we further examined the mediations of physician-patient communication, patient health information–seeking behavior, and the perceived quality of health information using the bootstrapping method (n=5000; 95% CI).

## Results

We sent the questionnaire to 560 participants and received 446 responses, 381 of which were valid. Accordingly, the response rate was 79.6% (446/560), and the validity rate was 85.4% (381/446). As shown in Table 1, 58.3% (222/381) of the participants were 20 to 40 years old, 53% (202/381) were female, and 51.4% (196/381) had at least a bachelor’s degree, all which accounted for more than half of the sample. Given that the participants were OHC users who tended to be young, female, and highly educated, our sample could be considered representative [71,72].

**Table 1:** Sample demographics (N=381).

Demographic characteristics		Value, n (%)
**Age (years)**		
	<20	18 (4.7)
	20-29	107 (28.1)
	30-39	115 (30.2)
	40-49	89 (23.4)
	50-59	48 (12.6)
	60 and above	4 (1.0)
**Gender**		
	Male	179 (47.0)
	Female	202 (53.0)
**Living area**		
	Urban	212 (55.6)
	Rural	169 (44.4)
**Education**		
	Junior middle school and below	19 (5.0)
	High school	50 (13.1)
	Junior college	116 (30.4)
	Bachelor’s degree	144 (37.8)
	Master’s degree	41 (10.8%)
	PhD	11 (2.9%)

The Cronbach α of eHealth literacy, physician-patient communication, internet health information–seeking behavior, perceived quality of internet health information, and patient adherence were .849, .898, .709, .905, and .771, respectively. Therefore, the reliability of the scales was acceptable [73]. The Kaiser-Meyer-Olkin value was .964, so the collected data could be used to conduct factor analysis [74]. This study adopted the composite reliability and the average variance extracted (AVE) to evaluate the validity of scales. As shown in Table 2, for each construct, the composite reliability was above 0.700 and the AVE was above 0.500, indicating an acceptable convergent validity [75]. Table 3 shows the correlations between constructs, and the discriminant validity was acceptable because the square root of AVE was greater than the correlations between other constructs and themselves [75].

**Table 2:** Composite reliability and average variance extracted.

Construct	CR^a^	AVE^b^	Sqrt^c^ AVE
eHealth literacy	0.916	0.522	0.723
Physician-patient communication in OHCs^d^	0.937	0.514	0.717
Internet health information–seeking behavior in OHCs	0.834	0.556	0.746
Perceived quality of internet health information in OHCs	0.943	0.506	0.712
Patient adherence	0.845	0.523	0.723

^a^CR: composite reliability.

^b^AVE: average variance extracted.

^c^Sqrt: square root.

^d^OHC: online health community.

**Table 3:** Correlations between constructs.

Construct	EHL^a^	PPC^b^	IHISB^c^	PQIHI^d^	PA^e^
EHL	1.000	—^f^	—	—	—
PPC	0.674	1.000	—	—	—
IHISB	0.632	0.690	1.000	—	—
PQIHI	0.665	0.681	0.681	1.000	—
PA	0.673	0.692	0.669	0.650	1.000

^a^EHL: eHealth literacy.

^b^PPC: physician-patient communication.

^c^IHISB: internet health information–seeking behavior.

^d^PQIHI: perceived quality of internet health information.

^e^PA: patient adherence.

^f^Not applicable.

In terms of the effects of demographic factors, results indicated that 4 control variables (age, gender, living area, and education level) were positively associated with patient adherence, and gender was positively associated with physician-patient communication and patients’ perceived quality of internet health information in OHCs. Specifically, older patients were more willing to take medication and maintain a healthy lifestyle in accordance with medical regimens and physicians’ advice than were younger patients; female patients were more likely to communicate with physicians through OHCs, perceive a higher quality of information, and be more willing to adhere to treatments and physicians than were male patients; patients who lived in rural areas were more compliant with physicians than those who lived in urban areas; highly educated patients were more willing to adhere to physicians than were patients with a low level of education. However, all path coefficients of relationships related to control variables were nearly zero, indicating weak effects. The multivariate coefficient of determination (*R*^2^) was used to calculate Cohen *ƒ*^2^ to further evaluate the size of the control variables’ effects [76] as listed in Table 4. We confirmed that the effect sizes of the control variables were so small that they could be considered insignificant.

**Table 4:** Multivariate coefficient of determination (R^2^) results.

Variables		*R* ^2^		Control variable effects		
		In	Out	∆*R*^2a^	*ƒ* ^b^	Effects
**Physician-patient communication**						
	Control variables	0.722	0.720	0.002	0.007	Insignificant
	eHealth literacy	0.677	0.004	0.673	2.084	Large
**Internet health information–seeking behavior**						
	Control variables	0.537	0.534	0.003	0.006	Insignificant
	eHealth literacy	0.537	0.003	0.534	1.153	Large
**Perceived quality of internet health information**						
	Control variables	0.677	0.676	0.001	0.003	Insignificant
	eHealth literacy	0.677	0.002	0.675	2.090	Large
**Patient adherence**						
	Control variables	0.617	0.613	0.004	0.010	Insignificant
	Physician-patient communication	0.617	0.571	0.046	0.120	Small
	Internet health information–seeking behavior	0.617	0.608	0.009	0.023	Small
	Perceived quality of internet health information	0.617	0.604	0.013	0.034	Small

^a^*∆R^2^*: *R*^2^_In_ – *R*^2^_Out_.

^b^ƒ^2^: Cohen ƒ^2^.

GoF was used to evaluate the fit between the research model and observed data [70,77] and was calculated as follows:



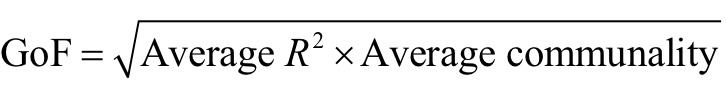



In this study, the GoF value was 0.578, which indicated that the fit between the data and the research model was good [78].

The magnitude and significance of path coefficients revealed that all 6 hypotheses were supported (Table 5). In particular, eHealth literacy was positively associated with physician-patient communication, internet health information–seeking behavior, and perceived quality of internet health information in OHCs. Physician-patient communication, internet health information–seeking behavior, and perceived quality of internet health information in OHCs were positively associated with patient adherence. We further analyzed the effect sizes of the independent variable and mediators, the results of which are presented in Table 4. We found that the effects of physician-patient communication, internet health information–seeking behavior, and the perceived quality of internet health information in OHCs on patient adherence were weak with small effect sizes, while the effects of eHealth literacy on physician-patient communication, internet health information–seeking behavior, and the perceived quality of internet health information in OHCs were strong with large effect sizes.

**Table 5:** Results of hypothesis testing.

Hypothesis	Path coefficient	*t*	*P* value
H1: Patients’ eHealth literacy is positively associated with physician-patient communication in OHCs.	0.849	46.484	<.001
H2: Patients’ eHealth literacy is positively associated with patients’ internet health information–seeking behavior in OHCs.	0.732	26.479	<.001
H3: Patients’ eHealth literacy is positively associated with patients’ perceived quality of internet health information in OHCs.	0.822	33.227	<.001
H4: Physician-patient communication in OHCs is positively associated with patient adherence.	0.429	4.423	<.001
H5: Patients’ internet health information–seeking behavior in OHCs is positively associated with patient adherence.	0.156	2.408	0.02
H6: Patients’ perception of internet health information quality is positively associated with patient adherence.	0.247	2.918	0.004

According to Baron and Kenny [79], we adopted a bootstrapping method (n=5000, 95% CI) to further examine the mediations. The results of *a*, *b*, *c*, and *c*’ are shown in Table 6, and the CIs (bias corrected) of *a* and *b* are shown in Table 7. Under the guidance of Wen and Ye [80], we could conclude that physician-patient communication and patient internet health information–seeking behavior in OHCs played partially mediating roles between eHealth literacy and patient adherence, and the mediating effect accounted for 30.07% and 13.25% of the total effect, respectively.

**Table 6:** Parameters of mediating effects.

*M*	*X*	*Y*	*a* (*P*)	*b* (*P*)	*c* (*P*)	c*’* (*P*)
PPC^a^	EHL^b^	PA^c^	0.849(<0.001)	0.291 (.005)	0.747 (<.001)	0.271 (.001)
IHISB^d^	EHL	PA	0.733(<0.001)	0.135 (.03)	0.747 (<.001)	0.271 (.001)
PQIHI^e^	EHL	PA	0.823(<0.001)	0.157 (.06)	0.747 (<.001)	0.271 (.001)

^a^PPC: physician-patient communication.

^b^EHL: eHealth literacy.

^c^PA: patient adherence.

^d^IHISB: internet health information–seeking behavior.

^e^PQIHI: perceived quality of internet health information.

**Table 7:** Confidence intervals (bias corrected).

*M*	*X*	*Y*	CIs (bias corrected) of *a*		CIs (bias corrected) of *b*	
			2.5%	97.5%	2.5%	97.5%
PPC^a^	EHL^b^	PA^c^	0.805	0.880	0.082	0.490
IHISB^d^	EHL	PA	0.668	0.776	0.013	0.260
PQIHI^e^	EHL	PA	0.770	0.863	–0.005	0.313

^a^PPC: physician-patient communication.

^b^EHL: eHealth literacy.

^c^PA: patient adherence.

^d^IHISB: internet health information–seeking behavior.

^e^PQIHI: perceived quality of internet health information.

## Discussion

### Principal Results

This study explored the association between patients’ eHealth literacy in OHCs and their adherence to treatment regimens and physicians’ suggestions, and it has theoretical contributions and practical implications for studies on OHCs, eHealth literacy, and patient adherence. We constructed a research model which clarified that patients’ eHealth literacy in OHCs can help improve their adherence by guiding their communication behavior with physicians, strengthening their health information–seeking behavior, and increasing their perceived quality of information in OHCs. This study enriches the theoretical research on OHCs, eHealth literacy, and patient adherence, and reduces the deficit in research related to improving patient adherence by strengthening patients’ eHealth literacy in OHCs in the Chinese context. In addition, this study adopted social cognitive theory in developing the research model for examining the relationship between eHealth literacy in OHCs and patient adherence, which enriches the application of social cognitive theory in the field of health behavioral psychology.

In terms of practical implications, our findings suggest that physician-patient communication, internet health information–seeking behavior, and patients’ perceived quality of internet health information are perspectives from which patient adherence can be enhanced by developing patients’ eHealth literacy in OHCs. First, among the 4 mediators, patients’ eHealth literacy was the strongest associated with physician-patient communication in OHCs, and physician-patient communication had the strongest association with patient adherence. Physician-patient communication plays a significant mediating role between eHealth literacy in OHCs and patient adherence, which suggests that physicians can encourage patients to communicate with them through OHCs in addition to offline treatments. OHCs can adopt some incentives to improve the possibility of patients’ participation in communication with physicians.

Second, in OHCs, the association between patients’ eHealth literacy and their perceived quality of internet health information was slightly weaker than that between eHealth literacy and physician-patient communication, but the effect size remained large. eHealth literacy can improve patients’ ability to appraise information quality accurately, ultimately encouraging them to adhere to physicians and the recommended treatments. This finding suggests that physicians and OHCs should focus not only on the actual information quality but also the perceived information quality. Evidence from Silver [81] reveals that some patients prefer to trust online sources rather than health care providers, but the limited level of eHealth literacy may make the treatment difficult. Previous studies suggest that eHealth literacy can be enhanced by considering several factors, such as education, computer skills, physical exercise [82], and medical experiences [81]. Therefore, physicians should aim to understand the level of patients’ eHealth literacy and their perception of quality during the communication process so as to better understand the possibility and extent of patients obtaining unsuitable health information. Physicians can then help patients improve the efficiency of treatment. OHCs should be developed to be convenient and easy to use, and to provide detailed guidelines that can aid patients’ usage. This may bring benefits to improving patients’ eHealth literacy and their perception of information quality in OHCs. In addition, to further improve OHCs, operators of OHCs should strengthen the management of information quality and acquire patients’ feedback on the perceived quality of health information. Third, eHealth literacy is associated with patient adherence through the mediation of internet health information–seeking behavior although the associations between eHealth literacy and internet health information–seeking behavior and between internet health information–seeking behavior and patient adherence were relatively weaker compared with the other 2 mediators. Schulz et al [12] found that patients with adequate eHealth literacy are more willing to seek health information online than those with inadequate eHealth literacy. The positive association between patients’ health information–seeking behavior and patient adherence is supported by Zhang et al [14]. However, some patients with limited eHealth literacy find it difficult to use or find suitable and reliable information through OHCs [56], thereby decreasing their intention to seek health information through OHCs. Given the advantages of OHCs, physicians can discuss the benefits and drawbacks of seeking information online with patients and encourage patients to obtain information by themselves. In addition, when patients find that their physicians have grasped the health information that the patients had themselves already acquired, they will consider their physicians to be professional and thus be more willing to adhere to their advice. Although patients may be unwilling to seek heath information online, they can perceive that their physicians are sincere and indeed concerned about them. Accordingly, discussion between physicians and patients is beneficial for strengthening patients’ trust in their physicians and improving the efficiency of treatment. Moreover, some medical professionals (eg, nurses) can actively discuss with patients the information obtained by patients through OHCs, thus possibly promoting patients’ additional use. Furthermore, the popularity of OHCs can be improved to increase the possibility of patients seeking health information through OHCs.

### Limitations

This study has several limitations and future directions. First, other mediators can be examined in future studies in addition to physician-patient communication, internet health information–seeking behavior, and perceived quality of internet health information. Second, the sample was a fairly specific one, and the results therefore cannot be generalized to a general or global context. To make these results more universal, further surveys in other countries could be conducted by comparing the similarities and differences between China and these countries. Third, our sampling did not capture the characteristics of the Chinese population because of the limitation of the research conditions, and the inclusion of Chinese census data can be considered in future studies. Fourth, in the research model, the 2 mediators, eHealth literacy and health information–seeking behaviour, may overlap. eHealth literacy includes the skill of seeking health information through OHCs, and eHealth literacy and health information–seeking behavior in OHCs may be related to each other. Fifth, the observed data were self-reported data, and we did not track the participants’ use of the internet. Sixth, we conducted the cross-sectional questionnaire investigation only once, so we failed to capture the dynamic changes in participants’ attitudes toward variables. Finally, the adherence measure we used has no recorded previous use. These issues can be addressed in future studies.

### Conclusions

This study aimed to identify the association between patients’ eHealth literacy in OHCs and patient adherence. The results revealed that eHealth literacy in OHCs is positively associated with patient adherence through the mediations of physician-patient communication, patient internet health information–seeking behavior, and the perceived quality of internet health information in OHCs. All 3 mediators are crucial for improving patient adherence. These findings suggest the following: physicians should encourage patients to seek health information and communicate with them through OHCs, understand the level of patients’ eHealth literacy and their perception of information quality, and help patients during the treatment to compensate for the lack of eHealth literacy; OHC operators should strengthen the management of information quality, make OHCs user friendly by providing detailed guidelines, and increase their popularity; OHC operators should improve the reliability of high-quality information provided to patients to decrease the gap between the actual and the perceived quality of health information.

## Appendix

Multimedia Appendix 1 Measurement instruments.

## Abbreviations

AVE: average variance extracted
GoF: goodness of fit
OHC: online health community
PLS: partial least squares
SEM: structural equation modelling
