# Supplementary material for: Association Between eHealth Literacy in Online Health Communities and Patient Adherence: Cross-sectional Questionnaire Study
Source: J Med Internet Res. 2021 Sep 13;23(9):e14908. doi: 10.2196/14908 (PMC8477298; doi:10.2196/14908)
Supplement: Multimedia Appendix 1 [file jmir_v23i9e14908_app1.doc]

## Multimedia Appendix 1

Table A1. Measurement Instruments.

| **Construct** | **Scale/Scoring** | **Items** |
| --- | --- | --- |
| eHealth Literacy | - 5-point Likert - Strongly disagree to strongly agree | 1. I know what health resources are available in online health communities. |
|  |  | 2. I know where to find helpful health resources in online health communities. |
|  |  | 3. I know how to find helpful health resources in online health communities. |
|  |  | 4. I know how to use online health communities to answer my health questions. |
|  |  | 5. I know how to use the health information I find from online health communities to help me. |
|  |  | 6. I have the skills I need to evaluate the health resources I find in online health communities. |
|  |  | 7. I can tell high-quality form low-quality health resources in online health communities. |
|  |  | 8. I feel confident in using information from online health communities to make health decisions. |
|  |  | **Supplementary items** |
|  |  | 9. How useful do you feel online health communities are in helping you in making decisions about your health? |
|  |  | 10. How important is it for you to be able to access health resources in online health communities? |
| Physician-patient Communication | - 5-point Likert - Strongly disagree to strongly agree | 1. In online health communities, physicians greeted me in a way that made me feel comfortable. |
|  |  | 2. In online health communities, physicians treated me with respect. |
|  |  | 3. In online health communities, physicians showed interest in my ideas about my health. |
|  |  | 4. In online health communities, physicians understood my main health concerns. |
|  |  | 5. In online health communities, physicians paid attention to me. |
|  |  | 6. In online health communities, physicians let me talk without interruptions. |
|  |  | 7. In online health communities, physicians gave me as much information as I wanted. |
|  |  | 8. In online health communities, physicians talked in terms I could understand. |
|  |  | 9. In online health communities, physicians checked to be sure I understood everything. |
|  |  | 10. In online health communities, physicians encouraged me to ask questions. |
|  |  | 11. In online health communities, physicians involved me in decisions as much as I wanted. |
|  |  | 12. In online health communities, physicians discussed next steps. |
|  |  | 13. In online health communities, physicians showed care and concern. |
|  |  | 14. In online health communities, physicians spent the right amount of time with me. |
| Perceived Quality of Health Information | - 5-point Likert - Strongly disagree to strongly agree | **Relevance:** |
|  |  | 1. For your health information needs, to what degree do you believe the health information provided by online health communities was applicable to your needs? |
|  |  | 2. For your health information needs, to what degree do you believe the health information provided by online health communities was related to your needs? |
|  |  | 3. For your health information needs, to what degree do you believe the health information provided by online health communities was pertinent to your needs? |
|  |  | 4. For your health information needs, to what degree do you believe the health information provided by online health communities was relevant to your needs? |
|  |  | **Understandability:** |
|  |  | 1. For your health information needs, to what degree do you believe the health information provided by online health communities was clear in meaning? |
|  |  | 2. For your health information needs, to what degree do you believe the health information provided by online health communities was easy to read? |
|  |  | 3. For your health information needs, to what degree do you believe the health information provided by online health communities was easy to comprehend? |
|  |  | 4. For your health information needs, to what degree do you believe the health information provided by online health communities was understandable? |
|  |  | **Adequacy:** |
|  |  | 1. For your health information needs, to what degree do you believe the health information provided by online health communities was sufficient? |
|  |  | 2. For your health information needs, to what degree do you believe the health information provided by online health communities was complete? |
|  |  | 3. For your health information needs, to what degree do you believe the health information provided by online health communities was adequate? |
|  |  | 4. For your health information needs, to what degree do you believe the health information provided by online health communities contained the necessary topics/categories? |
|  |  | **Usefulness:** |
|  |  | 1. For your health information needs, to what degree do you believe the health information provided by online health communities was informative? |
|  |  | 2. For your health information needs, to what degree do you believe the health information provided by online health communities was valuable? |
|  |  | 3. For your health information needs, to what degree do you believe the health information provided by online health communities was helpful? |
|  |  | 4. For your health information needs, to what degree do you believe the health information provided by online health communities was useful? |
| Internet Health Information Seeking | - 5-point Likert - Strongly disagree to strongly agree | 1. I am going to get new information and skills through online health communities to improve my health. |
|  |  | 2. I can get follow-up on new programs related to healthcare from online health communities. |
|  |  | 3. In online health communities, having a suitable relationship with others helps me to share information on illness prevention. |
|  |  | 4. In online health communities, I talk to my health care provider about how to perform self-monitoring even if I have difficulty understanding him or her. |
| Patient Adherence | - 5-point Likert - Strongly disagree to strongly agree | 1. I am following/did follow the doctor’s suggestions exactly. |
|  |  | 2. I am following/did follow the doctor’s drug/medication recommendations. |
|  |  | 3. I am following/did follow the doctor's orders, such as to stay in bed. |
|  |  | 4. I have returned or plan to return to the doctor on the schedule he/she suggested. |
|  |  | 5. I have had or plan to have the follow-up tests recommended by the doctor |
